# *trans*-Carbonyl­chloridobis[dicyclo­hex­yl(4-isopropyl­phen­yl)phosphane]rhodium(I) acetone monosolvate

**DOI:** 10.1107/S1600536811033447

**Published:** 2011-08-27

**Authors:** Sizwe Makhoba, Alfred Muller, Reinout Meijboom, Bernard Omondi

**Affiliations:** a Research Centre for Synthesis and Catalysis, Department of Chemistry, University of Johannesburg, PO Box 524, Auckland Park, Johannesburg 2006, South Africa

## Abstract

The title rhodium Vaska-type complex, *trans-*[RhCl{P(C_6_H_11_)_2_(C_6_H_4_-4-C_3_H_7_)_2_}_2_(CO)], crystallizes with an accompanying acetone solvent mol­ecule. The metal atom shows a distorted square-planar coordination environment with selected important geometrical parameters of Rh—P = 2.3237 (6) and 2.3253 (6) Å, Rh—Cl = 2.3724 (6) Å, Rh—C = 1.802 (2) Å, P—Rh—P = 173.42 (2)° and Cl—Rh—C = 179.13 (7)°. Effective cone angles for the two P atoms are 165 and 161°, respectively. Both isopropyl groups and the acetone mol­ecule are disordered with occupancy values of 0.523 (5):0.477 (5), 0.554 (8):0.446 (8) and 0.735 (4):0.265 (4), respectively. The crystal packing is stabilized by weak C—H⋯O and C—H⋯Cl contacts.

## Related literature

For examples of the packing disorder observed in Vaska-type complexes of Rh, Ir, Pd and Pt, see: Chen *et al.* (1991 ▶), Kuwabara & Bau (1994 ▶), Otto *et al.* (2000 ▶) and Otto (2001 ▶), respectively. For background to our investigation of the steric and electronic effects of group 15 ligands, see: Roodt *et al.* (2003 ▶); Muller *et al.* (2006 ▶, 2008 ▶). For the related synthesis of the *trans*-[IrCl(CO)(PPh_3_)_2_] complex, see: Collman *et al.* (1990 ▶). For background to cone angles, see Tolman (1977 ▶); Otto *et al.* (2000 ▶); Otto (2001 ▶). For background to the Cambridge Structural Database, see: Allen (2002 ▶).
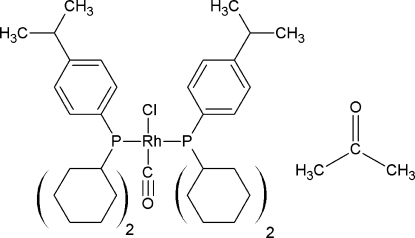


## Experimental

### 

#### Crystal data

[RhCl(C_21_H_33_P)_2_(CO)]·C_3_H_6_O
*M* *_r_* = 857.34
Tetragonal, 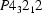

*a* = 16.0130 (15) Å
*c* = 35.557 (3) Å
*V* = 9117.4 (15) Å^3^
*Z* = 8
Mo *K*α radiation
μ = 0.54 mm^−1^
*T* = 100 K
0.34 × 0.24 × 0.14 mm

#### Data collection

Bruker APEX DUO 4K CCD diffractometer
Absorption correction: multi-scan (*SADABS*; Bruker, 2008 ▶) *T*_min_ = 0.838, *T*_max_ = 0.929
245346 measured reflections
11404 independent reflections
10089 reflections with *I* > 2σ(*I*)
*R*_int_ = 0.105

#### Refinement

*R*[*F* ^2^ > 2σ(*F* ^2^)] = 0.030
*wR*(*F* ^2^) = 0.059
*S* = 1.04
11404 reflections
544 parameters
31 restraints
H-atom parameters constrained
Δρ_max_ = 0.58 e Å^−3^
Δρ_min_ = −0.36 e Å^−3^
Absolute structure: Flack (1983 ▶), 4954 Friedel pairs
Flack parameter: −0.029 (17)

### 

Data collection: *APEX2* (Bruker, 2011 ▶); cell refinement: *SAINT* (Bruker, 2008 ▶); data reduction: *SAINT* and *XPREP* (Bruker, 2008 ▶); program(s) used to solve structure: *SIR97* (Altomare *et al.*, 1999 ▶); program(s) used to refine structure: *SHELXL97* (Sheldrick, 2008 ▶); molecular graphics: *DIAMOND* (Brandenburg & Putz, 2005 ▶); software used to prepare material for publication: *WinGX* (Farrugia, 1999 ▶).

## Supplementary Material

Crystal structure: contains datablock(s) global, I. DOI: 10.1107/S1600536811033447/zl2392sup1.cif

Structure factors: contains datablock(s) I. DOI: 10.1107/S1600536811033447/zl2392Isup2.hkl

Additional supplementary materials:  crystallographic information; 3D view; checkCIF report

## Figures and Tables

**Table 1:** Hydrogen-bond geometry (Å, °)

*D*—H⋯*A*	*D*—H	H⋯*A*	*D*⋯*A*	*D*—H⋯*A*
C131—H131⋯Cl	1.00	2.76	3.332 (2)	117
C221—H221⋯Cl	1.00	2.67	3.324 (2)	123
C224—H22*E*⋯O1^i^	0.99	2.70	3.359 (3)	124

Symmetry code: (i) 
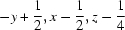
.

**Fig. 1.**
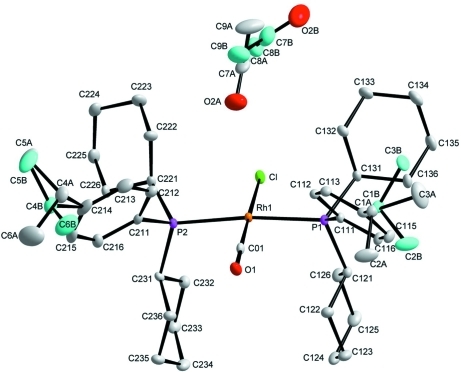
A view of the title compound showing the numbering scheme of atoms and displacement ellipsoids (drawn at a 30% probability level). Carbon atoms for the minor components of disordered parts are cyan colored. Hydrogen atoms have been omitted for clarity.

## supplementary crystallographic information

### Comment

Metal complexes with the general formula *trans*-[*M*(*X*)(Y)(*L*)_2_] (*M* = Ir(I), Rh(I), Pd(II), Pt(II); *X* = halogen or pseudo halogen; Y = carbonyl or halogen; *L* = tertiary group 15 ligand) often crystallize with the transition metal lying on a crystallographic centre of inversion resulting in a packing disorder of the *X* and Y moieties (Otto, 2001; Otto *et al.*, 2000; Chen *et al.*, 1991; Kuwabara & Bau, 1994). These Vaska type complexes are useful model complexes and provide several probing methods, *e.g.* NMR and IR, to investigate the steric and electronic effects of novel group 15 ligands (Roodt *et al.*, 2003; Muller *et al.*, 2006; Muller *et al.*, 2008). Presented here is the *trans-*[RhCl(CO){PCy_2_(C_6_H_4_-4-C_3_H_7_}_2_] complex, containing the first reported structure of this tertiary phosphane ligand (Allen, 2002; Cambridge Structural Database search, database accessed 10/06/2011).

The title compound (Fig. 1) crystallizes in the tetragonal space group *P*4_3_2_1_2 (*Z*=8). This results in molecules lying in general positions in the unit cell and hence no packing disorder of the Cl and CO moieties is observed. The asymmetric unit has one molecule of acetone situated centrally near the rhodium complex. The metal coordination environment is distorted. This is observed most prominently for the P2—Rh1—P1 angle of 173.42 (2)° (Rh1 is displaced 0.0698 (7) Å from the plane formed by P1, C01, P2 and Cl respectively; r.m.s. deviation of fitted atoms = 0.0575 Å). The Cl—Rh1—C01 angle of 179.18 (8)° appears unaffected as the chloride and carbonyl ligands are significantly less bulky than the tertiary phosphorus ligands. The distortion could possibly be attributed to packing effects induced by the isopropyl groups.

To determine the phosphorus ligand bulkiness, an adaptation of the well known Tolman cone angle model was used (Tolman, 1977). Instead of using a CPK model, the actual geometry from the crystal structure was taken to determine an 'effective cone angle' (Otto *et al.*, 2001). Two different cone angles of 165° and 161° were obtained for P1 and P2 respectively, indicating some flexibility of the substituents of this group 15 ligand. The difference in cone angles could possibly be attributed to interactions with the acetone solvate or packing effects induced by the isopropyl groups. Weak C—H···Cl/O interactions stabilize the crystal structure (see Table 1).

### Experimental

The synthesis of the Rh-Vaska complex was adapted from the synthesis of the *trans*-[IrCl(CO)(PPh_3_)_2_] complex (see Collman *et al.*, 1990). [RhCl_3_.xH_2_O] (50 mg, 0.24 mmol, using the anhydrous basis for the molecular weight calculation) was dissolved in dimethylformamide (DMF) and then heated under reflux for approximately one hour. During this time the colour changed from red to yellow, signalling the formation of the [Rh(µ-Cl)(CO)_2_]_2_ dimer. The solution was then allowed to cool to room temperature and dicyclohexyl-4-isopropylphenyl phosphane (159 mg; 0.5 mmol), dissolved in DMF (3 cm^3^) was added drop wise while stirring. This was followed by the addition of ice to the mixture and a precipitate formed. The solution was then centrifuged and the precipitate collected. This was then worked up by washing with water, extracting with dichloromethane (10 cm^3^), and drying with MgSO_4_ (*ca*. 4 g). The solution was then evaporated to give the Vaska complex in powder form. This powder was then checked for purity (see characterization below) and single crystals suitable for data collection were obtained by slow evaporation from an acetone solution.

Analytical data: IR (CHCl_3_), ν(CO) = 1965 cm^-1^. NMR: ^31^P {H} NMR (CDCl_3_, 400 MHz) = δ 35.1 (*d*,^1^*J*_Rh—P_ = 123 Hz, 2P).

### Refinement

All hydrogen atoms were positioned in geometrically idealized positions with C—H = 1.00 Å, 0.99 Å, 0.98 Å and 0.95 Å for methine, methylene, methyl and aromatic H atoms respectively. All hydrogen atoms were allowed to ride on their parent atoms with *U*_iso_(H) = 1.2*U*_eq_, except for methyl where *U*_iso_(H) = 1.5*U*_eq_ was utilized. The initial positions of methyl hydrogen atoms were located from a Fourier difference map and refined as a fixed rotor. The structure refined to a final Flack parameter of -0.029 (17). The highest residual electron density of 0.58 e.Å^-3^ is 0.65 Å from C6B representing no physical meaning. Initial refinement of the data showed large displacements of isopropyl groups and the acetone solvate. Subsequent refinement cycles involved treatment of these parts to disordered refinement procedures. Geometrical (SADI) restraints were applied to isopropyl groups bonded to the phenyl rings (C114—C1A/B and C214—C4A/B), as well as to 1,2- and some 1,3- bond distances (C1A/B—C2A/B and C1A/B—C3A/B, C4A/B—C5A/B and C4A/B—C6A/B, C5A/B—C6A/B) of the isopropyl moieties. Ellipsoid displacement (EADP) constraints were applied to disordered atom groups C1A/B, C2A/B, C4A/B, C5A/B and O2A/C9B. In the case of C6A/B ISOR had to be utilized. All restraints were applied with the default standard deviations, except for ISOR where 0.005 Å^2^ was used. In each case a free variable was connected to the disordered parts and refined to add to unity. For both isopropyl moieties, the free variables refined to almost 50:50 ratio's (0.523 (5):0.477 (5) and 0.554 (8):0.446 (8) for isopropyls attached to C114 and C214 respectively). The free variable for the acetone solvate refined to a ratio of 0.735 (4):0.265 (4). The final refined model shows slightly large carbon U_eq(max)_/U_eq(min)_ values, but was retained as these observations are primarily associated with the disordered parts.

### Crystal data

**Table:**

[RhCl(C_21_H_33_P)_2_(CO)]·C_3_H_6_O	*D*_x_ = 1.249 Mg m^−^^3^
*M**_r_* = 857.34	Mo *K*α radiation, λ = 0.71073 Å
Tetragonal, *P*4_3_2_1_2	Cell parameters from 9890 reflections
Hall symbol: P 4nw 2abw	θ = 2.3–27.4°
*a* = 16.0130 (15) Å	µ = 0.54 mm^−^^1^
*c* = 35.557 (3) Å	*T* = 100 K
*V* = 9117.4 (15) Å^3^	Block, yellow
*Z* = 8	0.34 × 0.24 × 0.14 mm
*F*(000) = 3648	

### Data collection

**Table:**

Bruker APEX DUO 4K CCD diffractometer	11404 independent reflections
graphite	10089 reflections with *I* > 2σ(*I*)
Detector resolution: 8.4 pixels mm^-1^	*R*_int_ = 0.105
φ and ω scans	θ_max_ = 28.4°, θ_min_ = 1.7°
Absorption correction: multi-scan (*SADABS*; Bruker, 2008)	*h* = −21→21
*T*_min_ = 0.838, *T*_max_ = 0.929	*k* = −21→18
245346 measured reflections	*l* = −47→46

### Refinement

**Table:**

Refinement on *F*^2^	Secondary atom site location: difference Fourier map
Least-squares matrix: full	Hydrogen site location: inferred from neighbouring sites
*R*[*F*^2^ > 2σ(*F*^2^)] = 0.030	H-atom parameters constrained
*wR*(*F*^2^) = 0.059	*w* = 1/[σ^2^(*F*_o_^2^) + (0.0171*P*)^2^ + 5.251*P*] where *P* = (*F*_o_^2^ + 2*F*_c_^2^)/3
*S* = 1.04	(Δ/σ)_max_ = 0.014
11404 reflections	Δρ_max_ = 0.58 e Å^−^^3^
544 parameters	Δρ_min_ = −0.36 e Å^−^^3^
31 restraints	Absolute structure: Flack (1983), 4954 Friedel pairs
Primary atom site location: structure-invariant direct methods	Flack parameter: −0.029 (17)

### Special details

**Table:**

Experimental. The intensity data was collected on a Bruker Apex DUO 4 K CCD diffractometer using an exposure time of 15 s/frame. A total of 3328 frames were collected with a frame width of 0.5° covering up to θ = 28.38° with 99.7% completeness accomplished.
Geometry. All e.s.d.'s (except the e.s.d. in the dihedral angle between two l.s. planes) are estimated using the full covariance matrix. The cell e.s.d.'s are taken into account individually in the estimation of e.s.d.'s in distances, angles and torsion angles; correlations between e.s.d.'s in cell parameters are only used when they are defined by crystal symmetry. An approximate (isotropic) treatment of cell e.s.d.'s is used for estimating e.s.d.'s involving l.s. planes.
Refinement. Refinement of *F*^2^ against ALL reflections. The weighted *R*-factor *wR* and goodness of fit *S* are based on *F*^2^, conventional *R*-factors *R* are based on *F*, with *F* set to zero for negative *F*^2^. The threshold expression of *F*^2^ > σ(*F*^2^) is used only for calculating *R*-factors(gt) *etc*. and is not relevant to the choice of reflections for refinement. *R*-factors based on *F*^2^ are statistically about twice as large as those based on *F*, and *R*- factors based on ALL data will be even larger.

### Fractional atomic coordinates and isotropic or equivalent isotropic displacement parameters (Å^2^)

**Table:**

	*x*	*y*	*z*	*U*_iso_*/*U*_eq_	Occ. (<1)
Rh1	0.296490 (11)	0.118541 (11)	0.226611 (5)	0.01316 (4)	
Cl	0.32709 (4)	0.14074 (4)	0.162099 (15)	0.02160 (12)	
C01	0.27320 (13)	0.10337 (14)	0.27571 (7)	0.0185 (4)	
O1	0.25881 (11)	0.09525 (11)	0.30693 (5)	0.0270 (4)	
P1	0.19418 (3)	0.22135 (3)	0.224492 (15)	0.01422 (11)	
P2	0.40800 (3)	0.02650 (4)	0.232680 (15)	0.01409 (12)	
C1A	−0.1068 (6)	0.1425 (10)	0.3267 (3)	0.0219 (12)	0.523 (5)
H1A	−0.1169	0.081	0.3246	0.026*	0.523 (5)
C2A	−0.0880 (4)	0.1633 (5)	0.36763 (16)	0.0478 (13)	0.523 (5)
H2A1	−0.0387	0.1321	0.3759	0.072*	0.523 (5)
H2A2	−0.136	0.1479	0.3833	0.072*	0.523 (5)
H2A3	−0.0774	0.2233	0.3701	0.072*	0.523 (5)
C3A	−0.1871 (4)	0.1884 (6)	0.3156 (2)	0.063 (3)	0.523 (5)
H3A1	−0.1769	0.2488	0.3155	0.094*	0.523 (5)
H3A2	−0.2313	0.1753	0.3337	0.094*	0.523 (5)
H3A3	−0.2045	0.1705	0.2904	0.094*	0.523 (5)
C4A	0.3139 (5)	−0.2813 (5)	0.3295 (2)	0.0332 (16)	0.554 (8)
H4A	0.2528	−0.2766	0.3351	0.04*	0.554 (8)
C5A	0.337 (2)	−0.3567 (14)	0.3058 (5)	0.052 (2)	0.554 (8)
H5A1	0.3975	−0.3598	0.3032	0.078*	0.554 (8)
H5A2	0.3111	−0.3514	0.2809	0.078*	0.554 (8)
H5A3	0.3162	−0.4075	0.3181	0.078*	0.554 (8)
C6A	0.3706 (7)	−0.2919 (5)	0.3661 (2)	0.081 (3)	0.554 (8)
H6A1	0.376	−0.2378	0.3788	0.122*	0.554 (8)
H6A2	0.426	−0.312	0.3587	0.122*	0.554 (8)
H6A3	0.3446	−0.3322	0.3832	0.122*	0.554 (8)
C1B	−0.1148 (6)	0.1476 (11)	0.3223 (3)	0.0219 (12)	0.477 (5)
H1B	−0.1062	0.094	0.3362	0.026*	0.477 (5)
C2B	−0.1380 (5)	0.2143 (5)	0.35103 (18)	0.0478 (13)	0.477 (5)
H2B1	−0.0907	0.2241	0.3679	0.072*	0.477 (5)
H2B2	−0.1863	0.1954	0.3657	0.072*	0.477 (5)
H2B3	−0.1521	0.2663	0.3379	0.072*	0.477 (5)
C3B	−0.1887 (3)	0.1353 (4)	0.29552 (17)	0.0281 (14)	0.477 (5)
H3B1	−0.1974	0.1864	0.2809	0.042*	0.477 (5)
H3B2	−0.2391	0.1229	0.3101	0.042*	0.477 (5)
H3B3	−0.1768	0.0887	0.2784	0.042*	0.477 (5)
C4B	0.3469 (6)	−0.2804 (7)	0.3325 (3)	0.0332 (16)	0.446 (8)
H4B	0.405	−0.2868	0.3424	0.04*	0.446 (8)
C5B	0.331 (3)	−0.3593 (17)	0.3116 (7)	0.052 (2)	0.446 (8)
H5B1	0.3759	−0.3686	0.2934	0.078*	0.446 (8)
H5B2	0.2775	−0.3553	0.2984	0.078*	0.446 (8)
H5B3	0.3291	−0.4061	0.3294	0.078*	0.446 (8)
C6B	0.2968 (7)	−0.2656 (5)	0.3655 (2)	0.062 (3)	0.446 (8)
H6B1	0.2928	−0.3171	0.3802	0.093*	0.446 (8)
H6B2	0.2407	−0.2478	0.3578	0.093*	0.446 (8)
H6B3	0.3227	−0.2218	0.3807	0.093*	0.446 (8)
C111	0.10397 (13)	0.20137 (14)	0.25449 (6)	0.0155 (4)	
C112	0.07887 (14)	0.11957 (15)	0.26091 (6)	0.0174 (5)	
H112	0.1091	0.0749	0.2498	0.021*	
C113	0.01013 (15)	0.10227 (15)	0.28339 (6)	0.0198 (5)	
H113	−0.006	0.0458	0.2873	0.024*	
C114	−0.03569 (15)	0.16564 (15)	0.30036 (6)	0.0199 (5)	
C115	−0.01049 (16)	0.24773 (16)	0.29398 (7)	0.0234 (5)	
H115	−0.0403	0.2922	0.3055	0.028*	
C116	0.05759 (14)	0.26562 (14)	0.27102 (7)	0.0200 (5)	
H116	0.0727	0.3221	0.2665	0.024*	
C121	0.23735 (14)	0.32196 (14)	0.24048 (6)	0.0187 (5)	
H121	0.1926	0.3653	0.2386	0.022*	
C122	0.26715 (17)	0.31755 (16)	0.28140 (7)	0.0261 (5)	
H12A	0.3082	0.2717	0.2841	0.031*	
H12B	0.2191	0.3052	0.298	0.031*	
C123	0.30747 (18)	0.40018 (16)	0.29338 (8)	0.0336 (7)	
H12C	0.2652	0.4452	0.2924	0.04*	
H12D	0.3274	0.3954	0.3196	0.04*	
C124	0.38058 (17)	0.42277 (16)	0.26785 (9)	0.0376 (7)	
H12E	0.4044	0.4771	0.2758	0.045*	
H12F	0.4248	0.3799	0.2702	0.045*	
C125	0.35206 (17)	0.42832 (16)	0.22722 (9)	0.0355 (7)	
H12G	0.4008	0.4398	0.2109	0.043*	
H12H	0.3123	0.4753	0.2245	0.043*	
C126	0.30983 (16)	0.34720 (16)	0.21440 (7)	0.0267 (6)	
H12I	0.3517	0.3018	0.2139	0.032*	
H12J	0.2883	0.3546	0.1885	0.032*	
C131	0.14855 (15)	0.24185 (14)	0.17773 (6)	0.0181 (5)	
H131	0.1961	0.2528	0.1602	0.022*	
C132	0.10437 (15)	0.16316 (15)	0.16354 (7)	0.0229 (5)	
H13A	0.1436	0.1154	0.1644	0.027*	
H13B	0.0566	0.1501	0.1802	0.027*	
C133	0.07292 (18)	0.17503 (18)	0.12330 (7)	0.0302 (6)	
H13C	0.1211	0.183	0.1062	0.036*	
H13D	0.0426	0.1242	0.1152	0.036*	
C134	0.01529 (18)	0.24991 (19)	0.12055 (8)	0.0333 (7)	
H13E	−0.036	0.2389	0.1353	0.04*	
H13F	−0.0012	0.2582	0.094	0.04*	
C135	0.05677 (17)	0.32905 (18)	0.13510 (7)	0.0309 (6)	
H13G	0.0158	0.3753	0.1348	0.037*	
H13H	0.1035	0.3444	0.1182	0.037*	
C136	0.09008 (16)	0.31768 (16)	0.17526 (7)	0.0245 (5)	
H13I	0.0426	0.3099	0.1928	0.029*	
H13J	0.1207	0.3686	0.183	0.029*	
C211	0.38831 (16)	−0.06260 (14)	0.26335 (6)	0.0184 (5)	
C212	0.30787 (17)	−0.09523 (15)	0.26457 (7)	0.0251 (5)	
H212	0.2647	−0.0697	0.2503	0.03*	
C213	0.2901 (2)	−0.16503 (17)	0.28653 (8)	0.0369 (7)	
H213	0.2348	−0.1864	0.2869	0.044*	
C214	0.3509 (2)	−0.20404 (17)	0.30784 (7)	0.0388 (8)	
C215	0.4306 (2)	−0.17083 (17)	0.30673 (7)	0.0347 (7)	
H215	0.4734	−0.196	0.3214	0.042*	
C216	0.44988 (17)	−0.10151 (16)	0.28472 (7)	0.0266 (6)	
H216	0.5054	−0.0806	0.2843	0.032*	
C221	0.44359 (15)	−0.02103 (15)	0.18819 (6)	0.0178 (5)	
H221	0.4465	0.0253	0.1694	0.021*	
C222	0.37912 (17)	−0.08287 (17)	0.17341 (7)	0.0269 (5)	
H22A	0.3234	−0.0562	0.1729	0.032*	
H22B	0.3763	−0.1319	0.1903	0.032*	
C223	0.40333 (18)	−0.1112 (2)	0.13367 (7)	0.0361 (7)	
H22C	0.3623	−0.1528	0.1246	0.043*	
H22D	0.4014	−0.0626	0.1165	0.043*	
C224	0.48995 (19)	−0.14917 (18)	0.13265 (8)	0.0337 (7)	
H22E	0.5053	−0.1616	0.1063	0.04*	
H22F	0.4897	−0.2025	0.1468	0.04*	
C225	0.55463 (18)	−0.09071 (18)	0.14967 (7)	0.0315 (6)	
H22G	0.5617	−0.0416	0.1331	0.038*	
H22H	0.609	−0.12	0.1511	0.038*	
C226	0.52968 (15)	−0.06103 (16)	0.18898 (7)	0.0231 (5)	
H22I	0.5294	−0.1091	0.2065	0.028*	
H22J	0.5711	−0.02	0.1982	0.028*	
C231	0.50106 (14)	0.07875 (15)	0.25216 (6)	0.0177 (4)	
H231	0.5474	0.037	0.2532	0.021*	
C232	0.52855 (15)	0.15070 (15)	0.22626 (8)	0.0233 (5)	
H23A	0.5418	0.1282	0.201	0.028*	
H23B	0.4821	0.191	0.2235	0.028*	
C233	0.60515 (16)	0.19557 (18)	0.24207 (7)	0.0302 (6)	
H23C	0.6534	0.157	0.2419	0.036*	
H23D	0.619	0.2438	0.2258	0.036*	
C234	0.59008 (17)	0.22631 (17)	0.28217 (8)	0.0320 (6)	
H23E	0.6418	0.2519	0.2921	0.038*	
H23F	0.5459	0.2695	0.282	0.038*	
C235	0.56370 (17)	0.15413 (18)	0.30758 (7)	0.0292 (6)	
H23G	0.6097	0.113	0.3093	0.035*	
H23H	0.5523	0.1755	0.3332	0.035*	
C236	0.48589 (15)	0.11165 (17)	0.29219 (6)	0.0236 (5)	
H23I	0.439	0.152	0.2919	0.028*	
H23J	0.4702	0.0646	0.3088	0.028*	
O2A	0.1421 (3)	0.9423 (2)	0.19253 (10)	0.0673 (12)	0.735 (4)
C7A	0.1229 (3)	0.9277 (3)	0.16044 (16)	0.0412 (12)	0.735 (4)
C8A	0.1729 (5)	0.9554 (5)	0.12697 (17)	0.0396 (14)	0.735 (4)
H8A1	0.2117	0.9996	0.1346	0.059*	0.735 (4)
H8A2	0.1352	0.9768	0.1075	0.059*	0.735 (4)
H8A3	0.2045	0.9079	0.117	0.059*	0.735 (4)
C9A	0.0460 (4)	0.8777 (4)	0.15197 (15)	0.0715 (19)	0.735 (4)
H9A1	0.0163	0.8655	0.1754	0.107*	0.735 (4)
H9A2	0.062	0.8252	0.1398	0.107*	0.735 (4)
H9A3	0.0095	0.9096	0.1352	0.107*	0.735 (4)
O2B	0.0178 (7)	0.9608 (7)	0.1191 (4)	0.079 (4)	0.265 (4)
C7B	0.0849 (11)	0.9422 (9)	0.1305 (4)	0.055 (4)	0.265 (4)
C8B	0.1651 (14)	0.9696 (15)	0.1112 (6)	0.052 (5)	0.265 (4)
H8B1	0.152	1.011	0.0918	0.079*	0.265 (4)
H8B2	0.192	0.921	0.0996	0.079*	0.265 (4)
H8B3	0.2029	0.9943	0.1298	0.079*	0.265 (4)
C9B	0.0960 (14)	0.8978 (12)	0.1642 (5)	0.0673 (12)	0.265 (4)
H9B1	0.0461	0.8643	0.1693	0.101*	0.265 (4)
H9B2	0.1052	0.9372	0.1849	0.101*	0.265 (4)
H9B3	0.1446	0.8609	0.1618	0.101*	0.265 (4)

### Atomic displacement parameters (Å^2^)

**Table:**

	*U*^11^	*U*^22^	*U*^33^	*U*^12^	*U*^13^	*U*^23^
Rh1	0.01277 (8)	0.01424 (8)	0.01245 (7)	0.00271 (7)	0.00052 (7)	0.00092 (7)
Cl	0.0220 (3)	0.0260 (3)	0.0169 (2)	0.0078 (2)	0.0037 (2)	0.0048 (2)
C01	0.0141 (10)	0.0176 (11)	0.0238 (11)	0.0057 (8)	0.0006 (9)	−0.0003 (10)
O1	0.0280 (10)	0.0342 (11)	0.0187 (8)	0.0082 (8)	−0.0009 (7)	0.0006 (8)
P1	0.0132 (3)	0.0138 (3)	0.0156 (2)	0.0022 (2)	0.0013 (2)	0.0018 (2)
P2	0.0141 (3)	0.0144 (3)	0.0137 (3)	0.0033 (2)	−0.0001 (2)	0.0007 (2)
C1A	0.0190 (18)	0.0212 (19)	0.026 (2)	−0.0022 (17)	0.0063 (16)	0.000 (2)
C2A	0.053 (3)	0.063 (4)	0.028 (2)	−0.029 (2)	0.022 (2)	−0.015 (2)
C3A	0.025 (3)	0.087 (6)	0.076 (5)	0.007 (4)	0.020 (3)	0.040 (5)
C4A	0.036 (5)	0.0222 (16)	0.041 (2)	−0.002 (4)	0.006 (4)	0.0106 (15)
C5A	0.062 (4)	0.023 (2)	0.070 (5)	−0.011 (2)	0.035 (6)	0.006 (3)
C6A	0.120 (5)	0.068 (4)	0.055 (4)	−0.028 (4)	−0.008 (4)	0.019 (3)
C1B	0.0190 (18)	0.0212 (19)	0.026 (2)	−0.0022 (17)	0.0063 (16)	0.000 (2)
C2B	0.053 (3)	0.063 (4)	0.028 (2)	−0.029 (2)	0.022 (2)	−0.015 (2)
C3B	0.013 (3)	0.035 (4)	0.036 (3)	−0.002 (2)	0.005 (2)	0.009 (3)
C4B	0.036 (5)	0.0222 (16)	0.041 (2)	−0.002 (4)	0.006 (4)	0.0106 (15)
C5B	0.062 (4)	0.023 (2)	0.070 (5)	−0.011 (2)	0.035 (6)	0.006 (3)
C6B	0.098 (5)	0.039 (4)	0.049 (4)	−0.022 (4)	0.030 (4)	0.009 (3)
C111	0.0139 (11)	0.0161 (10)	0.0166 (10)	0.0029 (9)	0.0000 (8)	0.0005 (9)
C112	0.0165 (11)	0.0163 (11)	0.0195 (11)	0.0004 (10)	−0.0016 (8)	−0.0016 (9)
C113	0.0205 (12)	0.0178 (12)	0.0209 (12)	−0.0030 (9)	−0.0013 (9)	−0.0003 (9)
C114	0.0178 (12)	0.0238 (13)	0.0181 (11)	−0.0036 (10)	0.0007 (9)	−0.0021 (10)
C115	0.0210 (13)	0.0206 (13)	0.0285 (14)	0.0021 (10)	0.0080 (11)	−0.0033 (10)
C116	0.0194 (11)	0.0141 (10)	0.0264 (13)	0.0010 (9)	0.0048 (10)	0.0020 (10)
C121	0.0171 (11)	0.0159 (11)	0.0232 (12)	0.0012 (9)	−0.0003 (9)	0.0010 (9)
C122	0.0281 (13)	0.0241 (14)	0.0260 (13)	−0.0015 (10)	−0.0024 (11)	−0.0043 (10)
C123	0.0318 (16)	0.0271 (15)	0.0418 (16)	−0.0017 (12)	−0.0069 (13)	−0.0126 (12)
C124	0.0234 (13)	0.0213 (13)	0.068 (2)	−0.0011 (11)	−0.0087 (15)	−0.0120 (13)
C125	0.0264 (14)	0.0218 (13)	0.0583 (19)	−0.0069 (11)	0.0057 (14)	0.0012 (14)
C126	0.0235 (13)	0.0226 (12)	0.0341 (14)	−0.0026 (11)	0.0064 (11)	0.0018 (11)
C131	0.0185 (11)	0.0190 (11)	0.0167 (11)	0.0043 (9)	−0.0008 (9)	0.0028 (9)
C132	0.0218 (13)	0.0259 (13)	0.0210 (12)	0.0001 (10)	−0.0047 (10)	0.0035 (10)
C133	0.0333 (15)	0.0368 (16)	0.0204 (13)	−0.0020 (12)	−0.0076 (11)	0.0022 (11)
C134	0.0254 (14)	0.0481 (18)	0.0264 (14)	−0.0014 (13)	−0.0078 (11)	0.0140 (13)
C135	0.0285 (14)	0.0363 (16)	0.0279 (14)	0.0082 (12)	−0.0014 (11)	0.0150 (12)
C136	0.0241 (13)	0.0234 (14)	0.0259 (12)	0.0075 (10)	0.0006 (10)	0.0058 (10)
C211	0.0259 (12)	0.0149 (10)	0.0143 (10)	0.0046 (10)	0.0021 (9)	−0.0009 (8)
C212	0.0321 (14)	0.0191 (12)	0.0243 (12)	−0.0009 (10)	0.0011 (11)	−0.0009 (9)
C213	0.0509 (18)	0.0256 (14)	0.0340 (15)	−0.0116 (14)	0.0071 (14)	0.0009 (12)
C214	0.077 (2)	0.0163 (12)	0.0235 (13)	0.0031 (15)	0.0115 (13)	0.0024 (11)
C215	0.056 (2)	0.0269 (15)	0.0211 (13)	0.0166 (14)	0.0005 (13)	0.0053 (11)
C216	0.0320 (14)	0.0267 (14)	0.0211 (12)	0.0085 (11)	0.0013 (10)	0.0015 (10)
C221	0.0194 (12)	0.0201 (12)	0.0140 (10)	0.0052 (9)	0.0001 (9)	0.0015 (9)
C222	0.0273 (13)	0.0334 (14)	0.0199 (12)	−0.0005 (12)	−0.0009 (11)	−0.0090 (10)
C223	0.0378 (16)	0.0497 (18)	0.0208 (13)	0.0098 (15)	−0.0035 (11)	−0.0149 (13)
C224	0.0454 (18)	0.0352 (16)	0.0205 (13)	0.0134 (13)	0.0011 (12)	−0.0077 (12)
C225	0.0325 (15)	0.0374 (16)	0.0246 (14)	0.0137 (12)	0.0034 (11)	−0.0052 (12)
C226	0.0192 (12)	0.0284 (14)	0.0217 (12)	0.0102 (10)	0.0003 (10)	−0.0027 (10)
C231	0.0131 (11)	0.0222 (13)	0.0177 (10)	0.0017 (8)	−0.0026 (9)	−0.0017 (9)
C232	0.0214 (12)	0.0223 (12)	0.0264 (12)	0.0009 (10)	0.0015 (11)	0.0020 (11)
C233	0.0208 (13)	0.0290 (14)	0.0406 (14)	−0.0049 (11)	0.0043 (11)	0.0000 (12)
C234	0.0230 (13)	0.0268 (14)	0.0462 (17)	−0.0044 (10)	−0.0034 (12)	−0.0097 (12)
C235	0.0245 (14)	0.0349 (16)	0.0283 (13)	−0.0004 (11)	−0.0063 (11)	−0.0098 (12)
C236	0.0212 (12)	0.0289 (13)	0.0207 (11)	−0.0014 (11)	−0.0007 (9)	−0.0065 (11)
O2A	0.093 (3)	0.058 (2)	0.050 (2)	−0.015 (2)	−0.015 (2)	0.0039 (17)
C7A	0.038 (3)	0.021 (2)	0.065 (3)	−0.0081 (18)	−0.003 (2)	−0.006 (2)
C8A	0.032 (3)	0.035 (3)	0.052 (4)	−0.003 (2)	−0.006 (3)	−0.007 (3)
C9A	0.069 (4)	0.086 (5)	0.060 (3)	−0.048 (3)	0.026 (3)	−0.032 (3)
O2B	0.047 (6)	0.060 (7)	0.129 (11)	−0.014 (5)	0.010 (7)	−0.008 (7)
C7B	0.072 (11)	0.040 (8)	0.054 (9)	0.010 (7)	0.010 (8)	0.000 (6)
C8B	0.040 (9)	0.053 (11)	0.064 (14)	0.001 (8)	−0.001 (11)	0.010 (10)
C9B	0.093 (3)	0.058 (2)	0.050 (2)	−0.015 (2)	−0.015 (2)	0.0039 (17)

### Geometric parameters (Å, °)

**Table:**

Rh1—C01	1.802 (2)	C131—C136	1.536 (3)
Rh1—P1	2.3237 (6)	C131—H131	1
Rh1—P2	2.3253 (6)	C132—C133	1.529 (3)
Rh1—Cl	2.3724 (6)	C132—H13A	0.99
C01—O1	1.141 (3)	C132—H13B	0.99
P1—C111	1.824 (2)	C133—C134	1.516 (4)
P1—C121	1.843 (2)	C133—H13C	0.99
P1—C131	1.846 (2)	C133—H13D	0.99
P2—C211	1.823 (2)	C134—C135	1.521 (4)
P2—C231	1.844 (2)	C134—H13E	0.99
P2—C221	1.846 (2)	C134—H13F	0.99
C1A—C114	1.519 (10)	C135—C136	1.535 (3)
C1A—C2A	1.524 (10)	C135—H13G	0.99
C1A—C3A	1.534 (11)	C135—H13H	0.99
C1A—H1A	1	C136—H13I	0.99
C2A—H2A1	0.98	C136—H13J	0.99
C2A—H2A2	0.98	C211—C212	1.391 (3)
C2A—H2A3	0.98	C211—C216	1.392 (3)
C3A—H3A1	0.98	C212—C213	1.393 (3)
C3A—H3A2	0.98	C212—H212	0.95
C3A—H3A3	0.98	C213—C214	1.383 (4)
C4A—C5A	1.517 (12)	C213—H213	0.95
C4A—C214	1.575 (8)	C214—C215	1.383 (4)
C4A—C6A	1.594 (10)	C215—C216	1.393 (4)
C4A—H4A	1	C215—H215	0.95
C5A—H5A1	0.98	C216—H216	0.95
C5A—H5A2	0.98	C221—C226	1.520 (3)
C5A—H5A3	0.98	C221—C222	1.524 (3)
C6A—H6A1	0.98	C221—H221	1
C6A—H6A2	0.98	C222—C223	1.534 (3)
C6A—H6A3	0.98	C222—H22A	0.99
C1B—C114	1.515 (11)	C222—H22B	0.99
C1B—C2B	1.524 (12)	C223—C224	1.515 (4)
C1B—C3B	1.532 (11)	C223—H22C	0.99
C1B—H1B	1	C223—H22D	0.99
C2B—H2B1	0.98	C224—C225	1.522 (4)
C2B—H2B2	0.98	C224—H22E	0.99
C2B—H2B3	0.98	C224—H22F	0.99
C3B—H3B1	0.98	C225—C226	1.529 (3)
C3B—H3B2	0.98	C225—H22G	0.99
C3B—H3B3	0.98	C225—H22H	0.99
C4B—C6B	1.440 (11)	C226—H22I	0.99
C4B—C5B	1.486 (14)	C226—H22J	0.99
C4B—C214	1.506 (10)	C231—C236	1.537 (3)
C4B—H4B	1	C231—C232	1.539 (3)
C5B—H5B1	0.98	C231—H231	1
C5B—H5B2	0.98	C232—C233	1.529 (3)
C5B—H5B3	0.98	C232—H23A	0.99
C6B—H6B1	0.98	C232—H23B	0.99
C6B—H6B2	0.98	C233—C234	1.528 (4)
C6B—H6B3	0.98	C233—H23C	0.99
C111—C112	1.389 (3)	C233—H23D	0.99
C111—C116	1.398 (3)	C234—C235	1.527 (4)
C112—C113	1.388 (3)	C234—H23E	0.99
C112—H112	0.95	C234—H23F	0.99
C113—C114	1.390 (3)	C235—C236	1.521 (3)
C113—H113	0.95	C235—H23G	0.99
C114—C115	1.394 (3)	C235—H23H	0.99
C115—C116	1.392 (3)	C236—H23I	0.99
C115—H115	0.95	C236—H23J	0.99
C116—H116	0.95	O2A—C7A	1.205 (6)
C121—C122	1.533 (3)	C7A—C9A	1.499 (6)
C121—C126	1.540 (3)	C7A—C8A	1.502 (8)
C121—H121	1	C8A—H8A1	0.98
C122—C123	1.533 (3)	C8A—H8A2	0.98
C122—H12A	0.99	C8A—H8A3	0.98
C122—H12B	0.99	C9A—H9A1	0.98
C123—C124	1.525 (4)	C9A—H9A2	0.98
C123—H12C	0.99	C9A—H9A3	0.98
C123—H12D	0.99	O2B—C7B	1.186 (18)
C124—C125	1.518 (4)	C7B—C9B	1.40 (2)
C124—H12E	0.99	C7B—C8B	1.52 (3)
C124—H12F	0.99	C8B—H8B1	0.98
C125—C126	1.534 (3)	C8B—H8B2	0.98
C125—H12G	0.99	C8B—H8B3	0.98
C125—H12H	0.99	C9B—H9B1	0.98
C126—H12I	0.99	C9B—H9B2	0.98
C126—H12J	0.99	C9B—H9B3	0.98
C131—C132	1.531 (3)		
			
C01—Rh1—P1	88.91 (7)	C134—C133—C132	111.1 (2)
C01—Rh1—P2	89.06 (7)	C134—C133—H13C	109.4
P1—Rh1—P2	173.42 (2)	C132—C133—H13C	109.4
C01—Rh1—Cl	179.13 (7)	C134—C133—H13D	109.4
P1—Rh1—Cl	90.47 (2)	C132—C133—H13D	109.4
P2—Rh1—Cl	91.49 (2)	H13C—C133—H13D	108
O1—C01—Rh1	178.8 (2)	C133—C134—C135	111.8 (2)
C111—P1—C121	105.66 (11)	C133—C134—H13E	109.3
C111—P1—C131	104.15 (10)	C135—C134—H13E	109.3
C121—P1—C131	105.72 (10)	C133—C134—H13F	109.3
C111—P1—Rh1	114.53 (7)	C135—C134—H13F	109.3
C121—P1—Rh1	110.17 (8)	H13E—C134—H13F	107.9
C131—P1—Rh1	115.75 (8)	C134—C135—C136	111.7 (2)
C211—P2—C231	105.67 (11)	C134—C135—H13G	109.3
C211—P2—C221	104.08 (10)	C136—C135—H13G	109.3
C231—P2—C221	105.04 (11)	C134—C135—H13H	109.3
C211—P2—Rh1	114.75 (8)	C136—C135—H13H	109.3
C231—P2—Rh1	111.58 (8)	H13G—C135—H13H	107.9
C221—P2—Rh1	114.77 (7)	C135—C136—C131	111.0 (2)
C114—C1A—C2A	112.8 (7)	C135—C136—H13I	109.4
C114—C1A—C3A	110.7 (9)	C131—C136—H13I	109.4
C2A—C1A—C3A	107.8 (8)	C135—C136—H13J	109.4
C114—C1A—H1A	108.5	C131—C136—H13J	109.4
C2A—C1A—H1A	108.5	H13I—C136—H13J	108
C3A—C1A—H1A	108.5	C212—C211—C216	118.1 (2)
C5A—C4A—C214	105.2 (14)	C212—C211—P2	118.22 (18)
C5A—C4A—C6A	103.4 (12)	C216—C211—P2	123.7 (2)
C214—C4A—C6A	105.6 (5)	C211—C212—C213	120.5 (3)
C5A—C4A—H4A	113.9	C211—C212—H212	119.7
C214—C4A—H4A	113.9	C213—C212—H212	119.7
C6A—C4A—H4A	113.9	C214—C213—C212	121.7 (3)
C114—C1B—C2B	114.6 (10)	C214—C213—H213	119.1
C114—C1B—C3B	110.5 (8)	C212—C213—H213	119.1
C2B—C1B—C3B	108.5 (9)	C213—C214—C215	117.4 (3)
C114—C1B—H1B	107.7	C213—C214—C4B	131.0 (5)
C2B—C1B—H1B	107.7	C215—C214—C4B	111.6 (5)
C3B—C1B—H1B	107.7	C213—C214—C4A	111.0 (4)
C1B—C2B—H2B1	109.5	C215—C214—C4A	131.6 (4)
C1B—C2B—H2B2	109.5	C214—C215—C216	121.8 (3)
H2B1—C2B—H2B2	109.5	C214—C215—H215	119.1
C1B—C2B—H2B3	109.5	C216—C215—H215	119.1
H2B1—C2B—H2B3	109.5	C211—C216—C215	120.4 (3)
H2B2—C2B—H2B3	109.5	C211—C216—H216	119.8
C1B—C3B—H3B1	109.5	C215—C216—H216	119.8
C1B—C3B—H3B2	109.5	C226—C221—C222	110.3 (2)
H3B1—C3B—H3B2	109.5	C226—C221—P2	115.98 (16)
C1B—C3B—H3B3	109.5	C222—C221—P2	110.75 (17)
H3B1—C3B—H3B3	109.5	C226—C221—H221	106.4
H3B2—C3B—H3B3	109.5	C222—C221—H221	106.4
C6B—C4B—C5B	116.8 (16)	P2—C221—H221	106.4
C6B—C4B—C214	111.3 (8)	C221—C222—C223	109.8 (2)
C5B—C4B—C214	114.0 (17)	C221—C222—H22A	109.7
C6B—C4B—H4B	104.4	C223—C222—H22A	109.7
C5B—C4B—H4B	104.4	C221—C222—H22B	109.7
C214—C4B—H4B	104.4	C223—C222—H22B	109.7
C4B—C5B—H5B1	109.5	H22A—C222—H22B	108.2
C4B—C5B—H5B2	109.5	C224—C223—C222	111.8 (2)
H5B1—C5B—H5B2	109.5	C224—C223—H22C	109.2
C4B—C5B—H5B3	109.5	C222—C223—H22C	109.2
H5B1—C5B—H5B3	109.5	C224—C223—H22D	109.2
H5B2—C5B—H5B3	109.5	C222—C223—H22D	109.2
C4B—C6B—H6B1	109.5	H22C—C223—H22D	107.9
C4B—C6B—H6B2	109.5	C223—C224—C225	111.5 (2)
H6B1—C6B—H6B2	109.5	C223—C224—H22E	109.3
C4B—C6B—H6B3	109.5	C225—C224—H22E	109.3
H6B1—C6B—H6B3	109.5	C223—C224—H22F	109.3
H6B2—C6B—H6B3	109.5	C225—C224—H22F	109.3
C112—C111—C116	118.1 (2)	H22E—C224—H22F	108
C112—C111—P1	119.38 (17)	C224—C225—C226	112.1 (2)
C116—C111—P1	122.51 (18)	C224—C225—H22G	109.2
C113—C112—C111	120.8 (2)	C226—C225—H22G	109.2
C113—C112—H112	119.6	C224—C225—H22H	109.2
C111—C112—H112	119.6	C226—C225—H22H	109.2
C112—C113—C114	121.5 (2)	H22G—C225—H22H	107.9
C112—C113—H113	119.2	C221—C226—C225	110.6 (2)
C114—C113—H113	119.2	C221—C226—H22I	109.5
C113—C114—C115	117.7 (2)	C225—C226—H22I	109.5
C113—C114—C1B	121.7 (7)	C221—C226—H22J	109.5
C115—C114—C1B	120.4 (7)	C225—C226—H22J	109.5
C113—C114—C1A	119.0 (6)	H22I—C226—H22J	108.1
C115—C114—C1A	123.2 (6)	C236—C231—C232	110.0 (2)
C116—C115—C114	121.1 (2)	C236—C231—P2	112.07 (16)
C116—C115—H115	119.5	C232—C231—P2	110.24 (15)
C114—C115—H115	119.5	C236—C231—H231	108.1
C115—C116—C111	120.7 (2)	C232—C231—H231	108.1
C115—C116—H116	119.6	P2—C231—H231	108.1
C111—C116—H116	119.6	C233—C232—C231	111.2 (2)
C122—C121—C126	110.4 (2)	C233—C232—H23A	109.4
C122—C121—P1	111.67 (16)	C231—C232—H23A	109.4
C126—C121—P1	109.05 (16)	C233—C232—H23B	109.4
C122—C121—H121	108.5	C231—C232—H23B	109.4
C126—C121—H121	108.5	H23A—C232—H23B	108
P1—C121—H121	108.5	C234—C233—C232	111.6 (2)
C121—C122—C123	110.8 (2)	C234—C233—H23C	109.3
C121—C122—H12A	109.5	C232—C233—H23C	109.3
C123—C122—H12A	109.5	C234—C233—H23D	109.3
C121—C122—H12B	109.5	C232—C233—H23D	109.3
C123—C122—H12B	109.5	H23C—C233—H23D	108
H12A—C122—H12B	108.1	C235—C234—C233	110.6 (2)
C124—C123—C122	111.3 (2)	C235—C234—H23E	109.5
C124—C123—H12C	109.4	C233—C234—H23E	109.5
C122—C123—H12C	109.4	C235—C234—H23F	109.5
C124—C123—H12D	109.4	C233—C234—H23F	109.5
C122—C123—H12D	109.4	H23E—C234—H23F	108.1
H12C—C123—H12D	108	C236—C235—C234	110.6 (2)
C125—C124—C123	110.5 (2)	C236—C235—H23G	109.5
C125—C124—H12E	109.6	C234—C235—H23G	109.5
C123—C124—H12E	109.6	C236—C235—H23H	109.5
C125—C124—H12F	109.6	C234—C235—H23H	109.5
C123—C124—H12F	109.6	H23G—C235—H23H	108.1
H12E—C124—H12F	108.1	C235—C236—C231	110.9 (2)
C124—C125—C126	111.5 (2)	C235—C236—H23I	109.5
C124—C125—H12G	109.3	C231—C236—H23I	109.5
C126—C125—H12G	109.3	C235—C236—H23J	109.5
C124—C125—H12H	109.3	C231—C236—H23J	109.5
C126—C125—H12H	109.3	H23I—C236—H23J	108
H12G—C125—H12H	108	O2A—C7A—C9A	120.3 (5)
C125—C126—C121	112.1 (2)	O2A—C7A—C8A	123.8 (5)
C125—C126—H12I	109.2	C9A—C7A—C8A	115.9 (5)
C121—C126—H12I	109.2	O2B—C7B—C9B	122.3 (17)
C125—C126—H12J	109.2	O2B—C7B—C8B	122.4 (14)
C121—C126—H12J	109.2	C9B—C7B—C8B	115.1 (17)
H12I—C126—H12J	107.9	C7B—C8B—H8B1	109.5
C132—C131—C136	110.5 (2)	C7B—C8B—H8B2	109.5
C132—C131—P1	109.49 (15)	H8B1—C8B—H8B2	109.5
C136—C131—P1	115.69 (17)	C7B—C8B—H8B3	109.5
C132—C131—H131	106.9	H8B1—C8B—H8B3	109.5
C136—C131—H131	106.9	H8B2—C8B—H8B3	109.5
P1—C131—H131	106.9	C7B—C9B—H9B1	109.5
C133—C132—C131	111.0 (2)	C7B—C9B—H9B2	109.5
C133—C132—H13A	109.4	H9B1—C9B—H9B2	109.5
C131—C132—H13A	109.4	C7B—C9B—H9B3	109.5
C133—C132—H13B	109.4	H9B1—C9B—H9B3	109.5
C131—C132—H13B	109.4	H9B2—C9B—H9B3	109.5
H13A—C132—H13B	108		
			
C01—Rh1—P1—C111	−34.86 (11)	C131—C132—C133—C134	−56.8 (3)
Cl—Rh1—P1—C111	145.75 (8)	C132—C133—C134—C135	55.4 (3)
C01—Rh1—P1—C121	84.08 (11)	C133—C134—C135—C136	−54.4 (3)
Cl—Rh1—P1—C121	−95.32 (8)	C134—C135—C136—C131	54.4 (3)
C01—Rh1—P1—C131	−156.08 (11)	C132—C131—C136—C135	−55.5 (3)
Cl—Rh1—P1—C131	24.52 (9)	P1—C131—C136—C135	179.40 (18)
C01—Rh1—P2—C211	38.16 (11)	C231—P2—C211—C212	156.74 (18)
Cl—Rh1—P2—C211	−142.51 (8)	C221—P2—C211—C212	−92.88 (19)
C01—Rh1—P2—C231	−81.99 (10)	Rh1—P2—C211—C212	33.4 (2)
Cl—Rh1—P2—C231	97.34 (8)	C231—P2—C211—C216	−25.4 (2)
C01—Rh1—P2—C221	158.68 (11)	C221—P2—C211—C216	85.0 (2)
Cl—Rh1—P2—C221	−21.99 (9)	Rh1—P2—C211—C216	−148.73 (17)
C121—P1—C111—C112	−153.34 (18)	C216—C211—C212—C213	−0.2 (3)
C131—P1—C111—C112	95.51 (18)	P2—C211—C212—C213	177.77 (19)
Rh1—P1—C111—C112	−31.89 (19)	C211—C212—C213—C214	0.3 (4)
C121—P1—C111—C116	27.7 (2)	C212—C213—C214—C215	0.3 (4)
C131—P1—C111—C116	−83.4 (2)	C212—C213—C214—C4B	−178.1 (6)
Rh1—P1—C111—C116	149.18 (17)	C212—C213—C214—C4A	−179.6 (4)
C116—C111—C112—C113	−0.6 (3)	C6B—C4B—C214—C213	−68.4 (12)
P1—C111—C112—C113	−179.56 (17)	C5B—C4B—C214—C213	66 (2)
C111—C112—C113—C114	−0.3 (3)	C6B—C4B—C214—C215	113.1 (8)
C112—C113—C114—C115	0.2 (4)	C5B—C4B—C214—C215	−112 (2)
C112—C113—C114—C1B	175.0 (6)	C6B—C4B—C214—C4A	−64.4 (19)
C112—C113—C114—C1A	−176.1 (5)	C5B—C4B—C214—C4A	70 (3)
C2B—C1B—C114—C113	157.2 (7)	C5A—C4A—C214—C213	98.3 (13)
C3B—C1B—C114—C113	−79.9 (12)	C6A—C4A—C214—C213	−152.7 (6)
C2B—C1B—C114—C115	−28.2 (12)	C5A—C4A—C214—C215	−81.5 (14)
C3B—C1B—C114—C115	94.7 (10)	C6A—C4A—C214—C215	27.5 (10)
C2B—C1B—C114—C1A	84 (7)	C5A—C4A—C214—C4B	−78 (3)
C3B—C1B—C114—C1A	−153 (9)	C6A—C4A—C214—C4B	30.5 (19)
C2A—C1A—C114—C113	110.5 (9)	C213—C214—C215—C216	−0.8 (4)
C3A—C1A—C114—C113	−128.6 (8)	C4B—C214—C215—C216	177.9 (5)
C2A—C1A—C114—C115	−65.5 (11)	C4A—C214—C215—C216	179.0 (5)
C3A—C1A—C114—C115	55.4 (9)	C212—C211—C216—C215	−0.3 (3)
C2A—C1A—C114—C1B	−138 (9)	P2—C211—C216—C215	−178.19 (19)
C3A—C1A—C114—C1B	−17 (7)	C214—C215—C216—C211	0.8 (4)
C113—C114—C115—C116	0.8 (4)	C211—P2—C221—C226	−70.5 (2)
C1B—C114—C115—C116	−174.1 (6)	C231—P2—C221—C226	40.3 (2)
C1A—C114—C115—C116	176.9 (5)	Rh1—P2—C221—C226	163.22 (16)
C114—C115—C116—C111	−1.7 (4)	C211—P2—C221—C222	56.12 (19)
C112—C111—C116—C115	1.5 (3)	C231—P2—C221—C222	166.96 (17)
P1—C111—C116—C115	−179.52 (19)	Rh1—P2—C221—C222	−70.12 (18)
C111—P1—C121—C122	62.27 (18)	C226—C221—C222—C223	−59.0 (3)
C131—P1—C121—C122	172.31 (17)	P2—C221—C222—C223	171.20 (18)
Rh1—P1—C121—C122	−61.95 (17)	C221—C222—C223—C224	56.9 (3)
C111—P1—C121—C126	−175.40 (16)	C222—C223—C224—C225	−53.6 (3)
C131—P1—C121—C126	−65.36 (18)	C223—C224—C225—C226	52.6 (3)
Rh1—P1—C121—C126	60.38 (17)	C222—C221—C226—C225	58.3 (3)
C126—C121—C122—C123	55.1 (3)	P2—C221—C226—C225	−174.83 (19)
P1—C121—C122—C123	176.66 (18)	C224—C225—C226—C221	−55.0 (3)
C121—C122—C123—C124	−57.7 (3)	C211—P2—C231—C236	−62.33 (19)
C122—C123—C124—C125	57.6 (3)	C221—P2—C231—C236	−172.02 (17)
C123—C124—C125—C126	−55.8 (3)	Rh1—P2—C231—C236	63.03 (18)
C124—C125—C126—C121	54.6 (3)	C211—P2—C231—C232	174.74 (16)
C122—C121—C126—C125	−53.9 (3)	C221—P2—C231—C232	65.04 (18)
P1—C121—C126—C125	−176.98 (18)	Rh1—P2—C231—C232	−59.91 (17)
C111—P1—C131—C132	−64.11 (18)	C236—C231—C232—C233	55.3 (3)
C121—P1—C131—C132	−175.22 (16)	P2—C231—C232—C233	179.41 (17)
Rh1—P1—C131—C132	62.54 (17)	C231—C232—C233—C234	−55.1 (3)
C111—P1—C131—C136	61.5 (2)	C232—C233—C234—C235	55.7 (3)
C121—P1—C131—C136	−49.6 (2)	C233—C234—C235—C236	−57.2 (3)
Rh1—P1—C131—C136	−171.83 (16)	C234—C235—C236—C231	58.4 (3)
C136—C131—C132—C133	56.8 (3)	C232—C231—C236—C235	−57.2 (3)
P1—C131—C132—C133	−174.60 (17)	P2—C231—C236—C235	179.80 (18)

### Hydrogen-bond geometry (Å, °)

**Table:**

*D*—H···*A*	*D*—H	H···*A*	*D*···*A*	*D*—H···*A*
C131—H131···Cl	1.00	2.76	3.332 (2)	117.
C221—H221···Cl	1.00	2.67	3.324 (2)	123.
C224—H22E···O1^i^	0.99	2.70	3.359 (3)	124.

Symmetry codes: (i) −*y*+1/2, *x*−1/2, *z*−1/4.
